# Remote ischemic conditioning: from experimental observation to clinical application: report from the 8th Biennial Hatter Cardiovascular Institute Workshop

**DOI:** 10.1007/s00395-014-0453-6

**Published:** 2014-12-02

**Authors:** Jack M. J. Pickard, Hans Erik Bøtker, Gabriele Crimi, Brian Davidson, Sean M. Davidson, David Dutka, Peter Ferdinandy, Rocky Ganske, David Garcia-Dorado, Zoltan Giricz, Alexander V. Gourine, Gerd Heusch, Rajesh Kharbanda, Petra Kleinbongard, Raymond MacAllister, Christopher McIntyre, Patrick Meybohm, Fabrice Prunier, Andrew Redington, Nicola J. Robertson, M. Saadeh Suleiman, Andrew Vanezis, Stewart Walsh, Derek M. Yellon, Derek J. Hausenloy

**Affiliations:** 1 The Hatter Cardiovascular Institute, University College London Hospital and Medical School, 67 Chenies Mews, London, WC1E 6HX UK; 2 Department of Cardiology, Aarhus University Hospital, Skejby, Aarhus N, Denmark; 3 Cardiology Department, Fondazione I.R.C.C.S. Policlinico San Matteo, Pavia, Italy; 4 Royal Free Hospital, London, UK; 5 Department of Medicine, University of Cambridge, Cambridge, CB2 0QQ UK; 6 Department of Pharmacology and Pharmacotherapy, Semmelweis University, Budapest, Hungary; 7 Pharmahungary Group, Szeged, Hungary; 8 CellAegis Devices Inc., Toronto, Canada; 9 Valld’Hebron University Hospital and Research Institute, Barcelona, Spain; 10 Neuroscience Physiology and Pharmacology, University College London, London, UK; 11 Universitaetsklinikum Essen, Essen, Germany; 12 Oxford University Hospitals NHS Trust, Headley Way, Oxford, UK; 13 Division of Medicine, University College London, London, UK; 14 SchulichSchool of Medicine and Dentistry, University of Western Ontario, Ontario, Canada; 15 Department of Anaesthesiology, Intensive Care Medicine and Pain Therapy, University Hospital Frankfurt, Frankfurt am Main, Germany; 16 Cardiology Department, L’UNAM Université, University of Angers, EA3860 Cardioprotection, Remodelage et Thrombose, University Hospital, Angers, France; 17 The Division of Cardiology, Department of Paediatrics, Hospital for Sick Children, University of Toronto, Toronto, Canada; 18 Neonatology, Institute for Women’s Health, University College London, London, WC1E 6HX UK; 19 Bristol Heart Institute Faculty of Medicine and Dentistry, University of Bristol, Bristol, UK; 20 Department of Cardiovascular Sciences, University of Leicester, Leicester, UK; 21 National University of Ireland, Galway, Ireland

**Keywords:** Ischemia, Organ protection, Remote ischemic conditioning, Reperfusion

## Abstract

In 1993, Przyklenk and colleagues made the intriguing experimental observation that ‘brief ischemia in one vascular bed also protects remote, virgin myocardium from subsequent sustained coronary artery occlusion’ and that this effect ‘…. may be mediated by factor(s) activated, produced, or transported throughout the heart during brief ischemia/reperfusion’. This seminal study laid the foundation for the discovery of ‘remote ischemic conditioning’ (RIC), a phenomenon in which the heart is protected from the detrimental effects of acute ischemia/reperfusion injury (IRI), by applying cycles of brief ischemia and reperfusion to an organ or tissue remote from the heart. The concept of RIC quickly evolved to extend beyond the heart, encompassing inter-organ protection against acute IRI. The crucial discovery that the protective RIC stimulus could be applied non-invasively, by simply inflating and deflating a blood pressure cuff placed on the upper arm to induce cycles of brief ischemia and reperfusion, has facilitated the translation of RIC into the clinical setting. Despite intensive investigation over the last 20 years, the underlying mechanisms continue to elude researchers. In the 8th Biennial Hatter Cardiovascular Institute Workshop, recent developments in the field of RIC were discussed with a focus on new insights into the underlying mechanisms, the diversity of non-cardiac protection, new clinical applications, and large outcome studies. The scientific advances made in this field of research highlight the journey that RIC has made from being an intriguing experimental observation to a clinical application with patient benefit.

## Introduction

Ischemic heart disease (IHD) maintains its unrelenting grip as the leading cause of death and disability worldwide. Therefore, novel therapeutic strategies are required to protect the heart against acute ischemia/reperfusion injury (IRI) to attenuate cardiomyocyte death, preserve cardiac function, prevent the onset of heart failure, and improve clinical outcomes in patients with IHD. In 1993, Przyklenk and colleagues [76] first demonstrated that applying cycles of brief ischemia and reperfusion to myocardium in the circumflex coronary artery territory protected remote virgin myocardium in the left anterior descending coronary artery territory. This intriguing observation extended the concept of direct ischemic preconditioning of the heart, initially described by Murry et al. [71] in 1986, to protect the heart at a distance or ‘remote ischemic conditioning’ (RIC). Over the last 20 years, the concept of RIC has evolved from being an experimental observation, whose underlying mechanisms continue to elude investigators, to a clinical application which offers the therapeutic potential to benefit patients with IHD (reviewed in [10, 31, 38–40]).

Yet many questions remain unanswered and several issues remain unresolved. The 8th Biennial Hatter Cardiovascular Institute Workshop, which was held at the University College London Hatter Cardiovascular Institute in the UK in April 2014, convened over 50 international investigators to discuss some of these questions and issues surrounding RIC. The focus of the Hatter Cardiovascular Institute (HCI) Workshop was on RIC induced by brief limb ischemia and reperfusion as this method of RIC has been the most clinically applicable strategy. The discussed topics included the mechanisms underlying RIC, non-cardiac RIC protection, the clinical application of RIC, and the potential for RIC to improve clinical outcomes.

### New insights into the mechanisms underlying RIC: why does it still elude us?

Despite intensive investigation over the last 20 years, the mechanisms underlying RIC remain unclear. The current paradigm divides the mechanistic pathway underlying RIC into three inter-related components as follows [10, 31, 38, 40]:

(1) Remote organ or tissue: in response to the RIC stimulus autacoids generated within the remote organ or tissue activate a local afferent neural pathway [62, 86, 95].

(2) The connecting pathway: the mechanistic pathway conveying the protective signal from the remote organ or tissue to the target organ or tissue has not been fully resolved. It has been shown to be dependent on both a humoral pathway (i.e. comprising blood-borne protective factor(s)) and a neural pathway to the remote organ or tissue.

(3) Target organ or tissue: the blood-borne protective factor(s) appear(s) to recruit intracellular signaling pathways from the remote organ or tissue which are known to mediate the protective effects induced by direct ischemic preconditioning and postconditioning.

#### What is the nature of the neural pathway underlying RIC?

Experimental and clinical studies have demonstrated that RIC protection is dependent on an intact neural pathway to the remote organ or tissue with local resection of the neural pathway abolishing RIC protection [27, 63]. However, the actual nature of the neural pathway in terms of its afferent, central, and efferent components remains unclear. The current paradigm has proposed that in response to the RIC stimulus, autacoids such as adenosine [23, 62, 86] and bradykinin [95] are produced in the remote organ or tissue resulting in the nitric oxide-dependant stimulation of local afferent sensory nerves. At the HCI Workshop, Kharbanda (Oxford, UK) presented unpublished human data investigating whether adenosine provides the ‘trigger’ for the limb RIC stimulus in IHD patients undergoing coronary angiography. Utilizing the human forearm model, they found that local arterial infusion of caffeine (a non-specific adenosine receptor antagonist) into the trigger arm blocked the beneficial effects of RIC on preventing ischemia-induced endothelial dysfunction, and inhibited the production of a cardioprotective plasma dialysate. Furthermore, the administration of an arterial infusion of adenosine into the femoral artery resulted in the production of a cardioprotective plasma dialysate in patients undergoing coronary angiography, confirming the findings in experimental animal studies that adenosine acted as a ‘trigger’ for limb RIC [86]. Most recent experimental data have suggested that the sensory arm of the neural pathway leading from the remote organ or tissue may be recruited by the activation of transient receptor potential vanilloid (TRPV) receptors, which are prevalent in unmyelinated small diameter (Aδ & C) sensory fibers [6, 47, 81]. Experimental studies have demonstrated that the activation of these fibers by topical capsaicin or nociceptive stimuli can recapitulate limb RIC cardioprotection [6, 47, 81].

However, the neural components of the pathway downstream of this sensory afferent neural pathway in the remote organ or tissue remain unclear. Jones et al. [47] found that cardioprotection elicited by peripheral nociception was blocked by spinal transection at T7 but not C7, suggesting that direct stimulation of cardiac nerves may be responsible for conveying the cardioprotective signal to the heart. In contrast to this study, and using an elegant experimental optogenetic approach, Gourine (London, UK) [64] has recently shown that the activity of the brainstem vagal preganglionic neuronsis required to mediate the protective effect of limb RIC on the heart, with their activation inducing powerful cardioprotection and their inhibition abrogating the beneficial effects of RIC [64]. To study the role of the efferent vagal pathway to limb RIC cardioprotection, Donato et al. [22] showed that resection of the vagal nerve and atropine abolished the MI-limiting effects of limb RIC in the rabbit heart and stimulation of the vagal nerve recapitulated limb RIC cardioprotection. However, dependency of limb RIC cardioprotection on the parasympathetic nervous system appears to preclude a role for a blood-borne cardioprotective factor.

Whether an efferent neural pathway is actually required to convey the cardioprotective signal to the heart or whether this is simply mediated by a blood-borne cardioprotective factor to the heart is not fully resolved. Kingma et al. [52] reported that neither the ganglionic blocker (hexamethonium) nor cardiac denervation abolished renal RIC protection of the canine heart. Similarly, Rassaf et al. [79] found that MI size reduction by limb RIC in the murine heart persisted despite femoral nerve resection (although the sciatic nerve was not resected in this model). Clearly, further studies are required to elucidate the details of the neural pathway underlying limb RIC cardioprotection.

#### What is the identity of the blood-borne cardioprotective factor?

The earliest experimental evidence for a blood-borne cardioprotective factor released by RIC was provided in 1999 by Dickson et al. [21], who demonstrated that the cardioprotective effect elicited by ischemic preconditioning of the heart and kidney in one rabbit could be transferred via whole blood transfusion to a non-preconditioned rabbit. Since then, a number of experimental studies have attempted to identify the blood-borne cardioprotective factor(s), resulting in a number of candidate factors being proposed including calcitonin gene-related peptide [87], opioids [73], endogenous cannabinoids [30], and hypoxia-inducible factor-1α (HIF-1α) [50].

Although the actual identity of the factor remains unclear, biochemical studies have suggested that the factor may be a peptide less than 30 kDa in size [58, 84]. Using proteomic analysis of plasma following RIC to identify the blood-borne cardioprotective factor(s) has been challenging. At the HCI Workshop, a number of novel candidates for the blood-borne cardioprotective factor(s) of RIC were proposed, each with varying degrees of experimental evidence: including (1) stromal-derived factor-1α or SDF-1α (S Davidson, London, UK) [19]; (2) exosomes (Giricz and Ferdinandy, Budapest, Hungary) [28]; nitrite (Heusch, Essen, Germany) [78, 79]; (3) microRNA-144 (Redington, Toronto, Canada) [60]; (4) HIF-1α (Prunier, Anger, France) [48]; and (5) Apolipoprotein a-I (Prunier) [41]. Of these, the most promising candidates for the blood-borne cardioprotective factor of RIC in terms of the available experimental evidence are probably SDF-1α, nitrite, and microRNA-144, as in these three cases limb RIC was demonstrated to elevate levels of the putative factor in the plasma, and blocking the factor also abolished the cardioprotective effect of RIC. However, these studies have failed to provide direct evidence that the factor secreted into the blood was actually responsible for the cardioprotective effect. Furthermore, it is important to note that none of these studies actually provided evidence that the production of the putative factor in response to RIC was dependent on an intact neural pathway to the limb, an important omission given that the blood-borne cardioprotective factor has been shown to be released downstream of the neural pathway (see next section).

#### How do the neural and humoral pathways interact to mediate RIC?

**Fig. 1:**
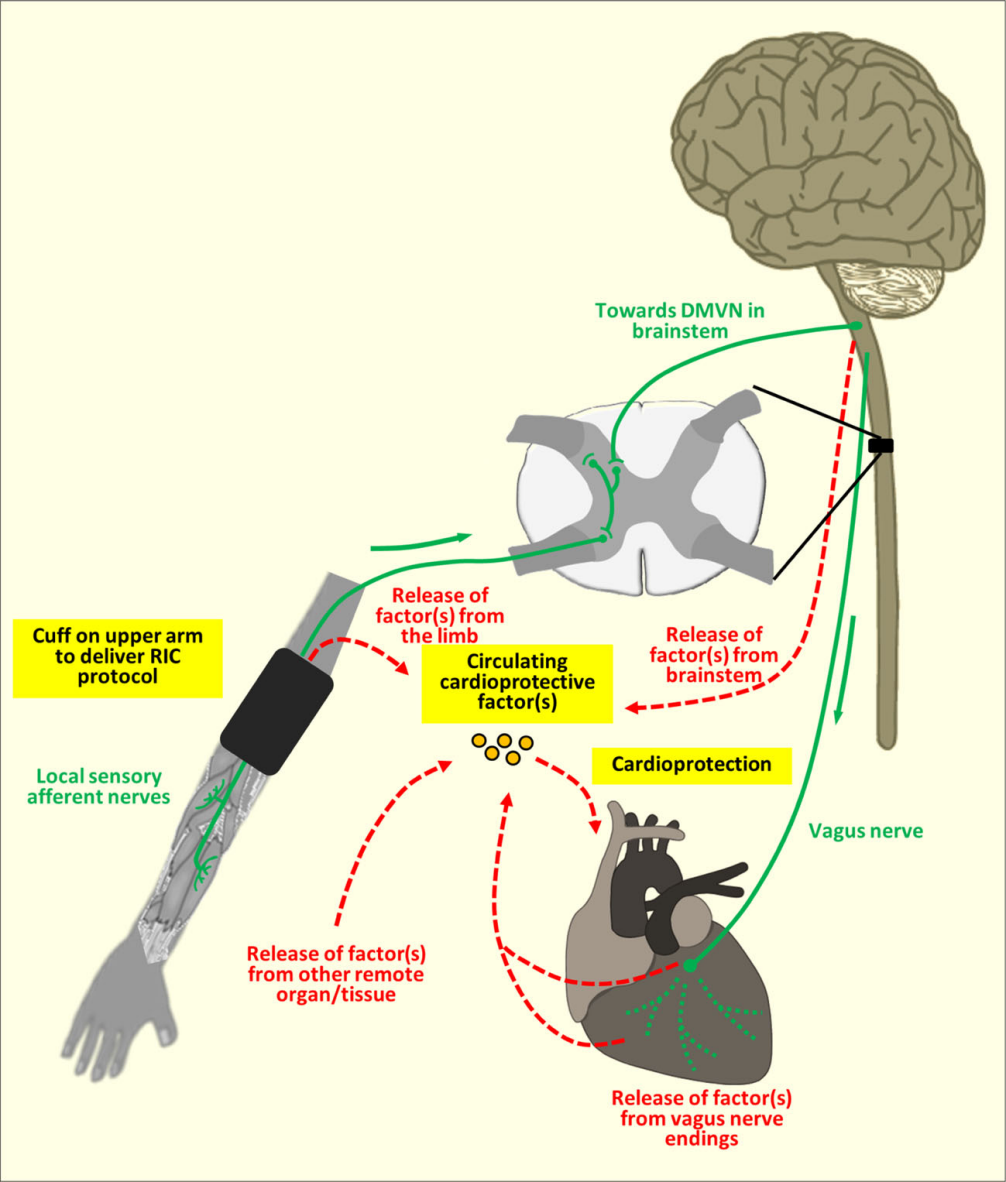
Connecting the limb to the heart in RIC. This figure shows the potential interplay between the neural pathway (*green solid lines*) and humoral pathway (*broken red lines*) in mediating RIC cardioprotection. Cycles of brief upper limb ischemia/reperfusion induced by inflation/deflation of a cuff placed on the upper arm produce the local release of autacoids, which then activate local sensory afferent neurons. One experimental study has shown the involvement of the neuronal activity in the brainstem dorsal motor vagal nucleus (DMVN) in RIC cardioprotection—this provides parasympathetic innervation of the left ventricle and other internal organs. A circulating blood-borne cardioprotective factor(s) is produced in response to the RIC stimulus downstream of the local sensory afferent neurons in the upper limb, but the actual source for its release is not currently known. Potential sites of release of the cardioprotective factor(s) include: (1) from the conditioned limb itself, (2) from the central nervous system (brainstem), (3) from pre-/post-ganglionic parasympathetic nerve endings within the heart (*broken green lines*); and (4) from a non-conditioned remote organ/tissue receiving parasympathetic innervation

The neural and humoral pathways underlying limb RIC have been known to interact to mediate the protective effect, but the actual nature of this relationship has not been clear until very recently (see Fig. 1 for a hypothetical scheme). Emerging studies from Redington’s and Botker’s research groups have begun to unravel the interplay between these two pathways in the setting of limb RIC. The major advance in this regard, has been facilitated by their use of an experimental model in which cardioprotective plasma dialysate harvested from animals or humans treated with limb RIC is demonstrated to reduce MI size in naïve animal hearts. Using this experimental model, they have been able to provide evidence showing that the blood-borne cardioprotective factor is produced downstream of the neural pathway. Redington’s group has shown that the cardioprotective plasma dialysate can be produced in animals and human volunteers in response to sensory neural stimulation of the limb using a number of different approaches including direct nerve stimulation [81], transcutaneous electrical nerve stimulation [68], electro-acupuncture [80] and even topical capsaicin [6, 81]. Botker’s group has demonstrated that diabetic patients with a peripheral sensory neuropathy in their upper limbs do not produce the cardioprotective plasma dialysate in response to limb RIC, when compared to diabetic patients with no sensory neuropathy [46]. Therefore, the combined evidence suggests that the blood-borne cardioprotective factor is most likely produced downstream of the neural pathway. But of course questions remain as to where along the neural pathway is the cardioprotective factor released into the blood stream, and which cell is actually responsible for its release.

#### Novel mediators of RIC cardioprotection in the heart

The current paradigm suggests that the cardioprotective signal initiated by limb RIC recruits signal transduction pathways (such as PI3K-Akt) in the target organ or tissue, which are known to be mediators of direct ischemic preconditioning and postconditioning [36, 37, 61]. In the HCI Workshop, data were presented implicating two novel mediators of limb RIC cardioprotection including aldehyde dehydrogenase-2 (ALDH-2) and phospho-myozenin-2. Kharbanda presented recent data showing in an animal MI model and human volunteers that the protective effect of limb RIC was abolished in the presence of an ALDH-2 inhibitor [13]. Interestingly, in support of a role for ALDH-2, human volunteers with a Glu504Lys polymorphism in ALDH-2 were found to be resistant to RIC protection against ischemia-induced endothelial dysfunction [13]. Further study is required to determine where in the mechanistic pathway ALDH-2 plays its mediatory role and to identify its downstream effectors. Suleiman (Bristol, UK) presented recent data investigating cardiac phosphoproteomics in the setting of limb RIC, demonstrating the phosphorylation of the cardiac sarcomeric protein, phospho-myozenin-2. These findings suggest that RIC may have functional effects on myocardial contractile function [1]. The importance of this to the cardioprotective effect induced by RIC remains to be investigated.

### Protecting non-cardiac organs by limb RIC

The key advantage of limb RIC as a therapeutic strategy is that it offers multi-organ protection against acute IRI. As such limb, RIC has been shown to be beneficial in a number of non-cardiac organs including the brain, the kidney, and the liver. In the HCI Workshop, a number of novel applications of RIC in non-cardiac protection were discussed.

#### Neuroprotection by RIC

It has been well established in the neuroprotection experimental literature that RIC can limit cerebral infarct size following an acute ischemic stroke [29]. At the HCI Workshop, Botker presented a recent clinical study investigating the effect of limb RIC in patients thrombolysed for an acute ischemic stroke—no clear benefit was found in terms of cerebral infarct size and functional recovery [44]. However, a small clinical study by Meng et al. [67] comprising 63 patients with prior stroke or transient ischemic accident demonstrated that RIC repeated twice daily for 300 days was able to reduce the recurrence of stroke and to improve functional recovery.

Cerebral IRI arising from perinatal hypoxic-ischemia, results in significant neonatal morbidity and long-term neurological impairment [59], despite the adoption of hypothermic neuroprotection in the developed world [5]. In this regard, N Robertson (London, UK) presented a recent study investigating the effect of limb RIC applied at the onset of reperfusion using a porcine model of neonatal cerebral hypoxia–ischemia. Limb RIC preserved cerebral white matter metabolism on magnetic resonance spectroscopy and reduced white matter cell death following transient global cerebral hypoxia–ischemia, suggesting that RIC may have therapeutic potential as a neuroprotective strategy for mitigating brain injury and improving outcomes in babies with birth asphyxia. This may have important implications in low resource countries where limb RIC could be used as a simple and low-cost neuroprotective intervention.

#### Renoprotection by RIC

Limb RIC has been investigated as a renoprotective strategy in several different clinical settings in which there is a risk of acute renal IRI [26]. In patients undergoing either cardiac bypass or major vascular surgery, acute renal IRI is a major determinant of acute kidney injury (AKI), a complication which occurs in 20–30 % of patients and which is associated with worse clinical outcomes. Several clinical studies have investigated a potential protective role of RIC on AKI in these surgical settings, but the results have been inconclusive [11, 77, 91]. The results of the large multicentre ERICCA [33] and RIPHeart [69] trials which are also investigating the effect of limb RIC on AKI should hopefully provide a definitive answer as to whether limb RIC is renoprotective in the setting of cardiac surgery.

Contrast-induced AKI (CI-AKI) is a significant cause of renal impairment in IHD patients undergoing coronary angiography and interventions, and one component of the injury is due to acute renal ischemic injury, and therefore a potential target for limb RIC [88]. Er et al. [24] have investigated in the Renal Protection Trial the effect of limb RIC on the incidence of CI-AKI in 100 high-risk patients undergoing coronary angiography and interventions who were pre-treated with intravenous normal saline and oral N-acetylcysteine—limb RIC reduced the incidence of CI-AKI from 40 to 12 %. The ERIC-CIN study in the UK is currently investigating whether the renoprotective effect of limb RIC is still present in 362 patients pre-treated with sodium bicarbonate prior to coronary angiography and procedures [7]. At the HCI Workshop, Crimi (Pavia, Italy) presented data investigating the effect of limb RIC on CI-AKI in STEMI patients treated by primary percutaneous coronary intervention (PPCI). In the original study, his team had already demonstrated a cardioprotective effect of limb RIC in this patient group with reduced enzymatic myocardial infarct size, and in this post hoc sub-group analysis they found that compared to control, limb RIC appeared to reduce the incidence of AKI in those STEMI patients with impaired renal function prior to PPCI [14, 15]. Finally, The EUROpean and Chinese cardiac and renal Remote Ischemic Preconditioning Study (EURO-CRIPS) trial will investigate both the renal and myocardial protective effects of limb RIC against CI-AKI and peri-procedural myocardial injury in 1,110 patients undergoing elective PCI, respectively [70].

Acute renal IRI sustained during pediatric renal transplantation is a critical determinant of graft function and clinical outcomes. MacAllister (London, UK) presented unpublished data from the REnal Protection Against Ischaemia–Reperfusion in transplantation (REPAIRISRCTN30083294) trial, a randomized double-blinded placebo-controlled trial of 400 living-donor renal transplant patients investigating the effect of limb RIC on renal graft function. He found that in those patients in whom limb RIC was administered to the donor and recipient, the estimated GFR at 6 months post-transplantation was increased compared to control, suggesting limb RIC to be a potential therapeutic strategy for preserving renal graft function post-transplantation.

#### Liver protection by RIC

B Davidson (London, UK) has been investigating in pre-clinical studies the protective effect and mechanisms underlying hepatic protection against acute IRI induced by limb RIC [2, 3, 49]. In the HCI Workshop, data were presented translating this therapeutic approach into the clinical setting, with a small study of 16 patients showing that limb RIC reduced the release of liver enzymes following liver resection surgery (ClinicalTrials.gov Identifier: NCT007965880). The ongoing Remote Ischaemic PreCOnditioning in Liver Transplant (RIPCOLT) study is currently investigating the efficacy of limb RIC in 40 liver transplant patients on liver protection and graft and patient survival.

### Novel clinical applications of RIC to protect the heart

The first clinical study to demonstrate the clinical application of limb RIC was by Redington and colleagues in 2006 who reported beneficial effects with this intervention in children undergoing corrective cardiac surgery [12] (Table 1). Since then limb RIC has been shown to attenuate acute myocardial IRI in a number of different clinical settings including cardiac bypass surgery [35, 89], major vascular surgery [4], elective PCI [43], and more recently STEMI patients treated by PPCI [8, 15, 75, 82, 94] (Table 1). In the HCI Workshop, Walsh (Galway, Ireland) presented details of the forthcoming Preconditioning Shields Against Vascular Events in Surgery (SAVES) trial (ClinicalTrials.gov Identifier:NCT01691911) which will investigate the effect of limb RIC on peri-operative myocardial injury in 400 patients undergoing major vascular surgery.

**Table 1:** Major clinical studies investigating the cardioprotective effects of limb RIC

Study	*N* number	RIC protocol	Results	Comments
Cardiac bypass surgery				
Cheung et al. [12]	37 children	4 × 5 min cycles of leg cuff	Smaller peak Trop T, less inotrope support and lower airway pressures	First study to test effect of limb RIC in the clinical setting
Hausenloy et al. [35]	53 adults	4 × 5 min cycles of arm cuff	43 % less 72 h AUC Trop T	First study to test effect of limb RIC in CABG surgery
Candilio et al. [11]	180 adults	4 × 5 min cycles of arm cuff	27 % less 72 h AUC Trop T. 54 % Less AF 48 % Less AKI and 1 day shortened ICU stay	First study to test effect of limb RIC on short-term outcomes following CABG surgery
Thielmann et al. [90]	329 adults	3 × 5 min cycles of arm cuff	21 % less 72 h AUC Trop I. 73 % reduction in all-cause mortality	First study to test effect of limb RIC on long-term outcomes following CABG surgery
Meybohm et al. [69] RIPHeart	1,403 adults recruitment completed	4 × 5 min cycles of arm cuff	Primary endpoint of death, non-fatal MI, stroke, AKI until hospital discharge Follow-up for 12 months.	First multi-centerstudy which will test effect of limb RIC on hard clinical endpoints following cardiac surgery Results available Mar 2,015
Hausenloy et al. [33] ERICCA	1,610 recruitment completed	4 × 5 min cycles of arm cuff	Primary endpoint of death, non-fatal MI, revascularization, stroke at 12 months	First multi-center study which will test effect of RIC on long-term clinical endpoints at 12 months Results available Mar 2015
Percutaneous coronary intervention (PCI)				
Hoole et al. [43] CRISP	242 adults	3 × 5 min cycles of arm cuff	63 % reduction in median Trop I	First study to test effect of RIC in PCI
Davies et al. [20]	192 adults	3 × 5 min cycles of arm cuff	42 % reduction in all-cause mortality, non-fatal MI, TIA or stroke, HHF at 6 years	First study to test effect of RIC on long-term clinical outcomes following PCI
ST-segment elevation myocardial infarction (STEMI)				
Botker et al. [8] CONDI-1	142 adults PPCI	4 × 5 min cycles of arm cuff prior to PPCI	36 % increase in myocardial salvage	First study to test effect of RIC in PPCI-treated STEMI patients
Crimi et al. [15] RemPostCond	96 adults PPCI	3 × 5 min cycles of thigh cuff at the time of PPCI	20 % reduction of MI size (AUC CK-MB) Reduction in myocardial edema on T2-weighted cardiac MRI	First study to show reduction in MI size and myocardial edema in anterior STEMI patients undergoing PPCI
Hausenloy et al. ERIC-LYSIS (clinicaltrials.gov identifier: NCT02197117)	519 adults thrombolysis	4 × 5 min cycles of arm cuff prior to thrombolysis	Primary endpoint of enzymatic MI size reduced by 17 %	Only study to test effect of RIC in thrombolysed STEMI patients
Sloth et al. [85]	251 adults PPCI	4 × 5 min cycles of arm cuff prior to PPCI inflation/deflation	51 % reduction in all-cause mortality, non-fatal MI, TIA or stroke, HHF at 3.8 years	First study to test effect of RIC on long-term outcomes following PPCI
Botker et al. CONDI-2 Hausenloy et al. ERIC-PPCI ClinicalTrials.gov Identifier:NCT01857414	4,300 adults PPCI Ongoing	4 × 5 min cycles of arm cuff inflation/deflation prior to PPCI	Primary endpoint of cardiac death and HHF at 12 months	Collaboration between UK and Denmark. This will be the first study to test effect of RIC on long-term clinical outcomes following PPCI

*AF* atrial fibrillation, *AKI* acute kidney injury, *AUC* area under curve, *CABG* coronary artery bypass graft, *HHF* hospitalization for heart failure, *MI* myocardial infarction, *MRI* magnetic resonance imaging, *PPCI* primary percutaneous coronary intervention, *RIC* remote ischemic conditioning, *TIA* transient ischemic accident

In the HCI Workshop, several novel applications of limb RIC for protecting the heart were discussed. Garcia-Dorado (Barcelona, Spain) presented unpublished data demonstrating the synergistic effect of limb RIC with either exenatide or glucose–insulin–potassium therapy administered at the time of reperfusion in terms of MI reduction in an in vivo porcine model of acute IRI. The concept of combining therapies which have a potential synergistic cardioprotective effect has not yet been tested in the clinical setting and it may actually be a more effective therapeutic strategy than using a mono therapy approach.

Limb RIC has already been shown to reduce MI size in STEMI patients treated by PPCI (Table 1). However, in developing countries in which PPCI is not readily available, STEMI patients are still reperfused by thrombolytic therapy—whether RIC is cardioprotective in this setting is not known. In the HCI Workshop, Hausenloy & Yellon (London, UK) presented unpublished results of the ERIC-LYSIS study (ClinicalTrials.gov Identifier:NCT02197117), a 519 STEMI patient multi-center clinical trial in the Island of Mauritius, showing that limb RIC initiated on arrival at the hospital prior to thrombolysis, reduced serum enzymatic MI size by 17 %. A large clinical outcome study is now planned to investigate whether limb RIC can reduce cardiac death and hospitalization for heart failure at 12 months in thrombolysed STEMI patients (the ERIC-LYSIS 2 trial).

The effect of RIC on exercise capacity in patients with heart failure has recently been investigated by Redington and colleagues [66]. Although they found no improvement in oxygen consumption with RIC when compared to sham, they did observe that plasma dialysate from both sham and RIC patients reduced murine MI size compared to plasma dialysate from historical healthy controls, suggesting heart failure patients, irrespective of RIC or sham intervention, may be subjected to a permanent chronic preconditioning stimulus per se [66].

Most previous clinical studies have investigated the cardioprotective effects of a single limb RIC stimulus targeted against an acute episode of IRI. Whether repeated episodes of limb RIC, applied as a chronic therapeutic intervention, are also beneficial has been recently investigated. An experimental study has reported that repeating RIC daily for 28 days prevented adverse post-MI left ventricle (LV) remodeling in the rat heart [93]. The mechanism for this beneficial effect is not clear but may relate to RIC-mediated attenuation of the immune, inflammatory and apoptotic response to MI. The concept of daily RIC is already being tested in the clinical setting in several clinical studies. Vanezis (Leicester, UK) presented details of the ongoing Daily REmote Ischaemic Conditioning following Acute Myocardial Infarction (DREAM, ClinicalTrials.gov Identifier: NCT01664611) trial in the UK, which is exploring the effect of daily RIC initiated after PPCI and continued for 4 weeks in 72 STEMI patients presenting with impaired LV ejection fraction (EF < 45 %)—primary endpoint of >5 % improvement in LVEF at 4 weeks post-MI. In Canada, the Chronic Remote Ischemic Conditioning to Modify Post-MI Remodeling (CRIC-RCT;ClinicalTrials.gov Identifier:NCT01817114) trial in Canada is testing the effect of repeating RIC daily for 28 days on the change from baseline in LV end diastolic volume at 28 days by cardiac MRI in 82 STEMI patients treated by PPCI. Finally, in the CONDI-HF study (ClinicalTrials.gov Identifier:NCT02248441), Botker and colleagues are currently investigating the effect of daily RIC in 50 chronic heart failure patients using LV ejection fraction assessed by cardiac MRI as the primary endpoint.

Chronic renal failure patients treated by haemodialysis have a significantly increased risk of cardiovascular morbidity and mortality. These patients experience repeated bouts of acute myocardial ischemia and stunning every time they have haemodialysis leading to chronic impairment of LV systolic function, resulting in *de novo* and recurrent heart failure with a 2-year mortality rate of 51 % [9]. At the HCI Workshop, McIntyre (Ontario, Canada) presented data investigating the potential cardioprotective benefit of RIC in this patient group. They found that limb RIC administered prior to haemodialysis prevented ST-segment depression and attenuated myocardial stunning compared to control, suggesting a potential cardioprotective effect of RIC on myocardial function in patients with chronic kidney failure [16]. Interestingly, it has been observed that haemodialysis patients with arteriovenous fistula experience fewer complications and lower mortality when compared to patients with alternative forms of vascular access [74]. Whether the beneficial effect of having arteriovenous fistula is inadvertently limb preconditioning the patient by inducing episodes of limb ischemia was raised as a possibility by McIntyre [54].

The majority of published clinical studies investigating the efficacy of limb RIC have used a manual blood pressure cuff to apply the RIC protocol. However, there is currently an automated cuff device available for delivering the limb RIC protocol. Ganske (CellAegis, Toronto, Cananda) presented the AutoRIC device which is able to deliver a standard limb RIC protocol (four 5 min cycles of upper arm cuff inflation/deflation) with a single push of a button, facilitating the delivery of limb RIC in clinical trials, especially where it is proposed as a potential chronic therapy.

### Why the neutral clinical RIC studies?

A number of clinical studies have failed to find any beneficial effects of limb RIC in patients undergoing PCI [45], CABG [51] and vascular surgery [92]; these include some large clinical trials conducted in pediatric [65] and adult cardiac surgery [42, 77]. Recent meta-analyses have for the most part reported beneficial effects with limb RIC in terms of reducing myocardial injury in the settings of cardiac bypass surgery [17] and PCI [18].

The one setting in which the effect of RIC has been predominantly positive is in STEMI patients treated by PPCI with five proof-of-concept studies reporting cardioprotective effects with limb RIC applied at the time of PPCI [8, 15, 75, 82, 94]. Several review articles have been published analyzing the potential reasons underlying the failure to translate cardioprotection into the clinical setting [32, 34, 72, 83]. At the HCI Workshop, some of these factors were discussed—they relate to patient selection, the RIC stimulus (the optimal stimulus remains unclear), the blinding of the RIC stimulus, the study design and choice of measured endpoints, confounding factors (such as age, diabetes, hyperlipidemia which may interfere with cardioprotection), and concomitant medications (such as volatile anesthetics, nitrates, statins which also interfere with cardioprotection) [25, 32, 34, 72]. Heusch presented a retrospective analysis of the Essen RIC trial on CABG patients [90], and identified anesthesia [55, 56], age, duration of index ischemia and sulphonylurea treatment of diabetics [57], but not use of nitroglycerine during surgery [53] as potential confounders.

### Improving clinical outcomes with limb RIC—Will it change clinical practice?

Most of the published clinical studies have established that limb RIC can limit myocardial injury in PCI, CABG and STEMI patients (Table 1). In the HCI Workshop, Hausenloy presented the results of a clinical study reporting that limb RIC could reduce the incidence of post-operative atrial fibrillation, acute kidney injury, and it could shorten ITU stay in patients undergoing CABG plus or minus valve surgery, suggesting some benefit on short-term clinical outcomes post-surgery [11]. Whether limb RIC can actually improve long-term clinical outcomes in these clinical settings remains unknown. In this regard, Botker, Heusch, and Dutka (Cambridge, UK) presented data at the HCI Workshop suggesting that limb RIC may improve long-term clinical endpoints in STEMI [85], CABG surgery [90] and elective PCI [20] patients, respectively, although none of these studies were prospectively designed or powered to investigate the effect of limb RIC on long-term clinical outcomes (Table 1). Meybohm and Hausenloy presented the forthcoming RIPHEART [69] and ERICCA [33] trials, respectively, which have been powered to investigate whether limb RIC can improve clinical outcomes at their primary endpoint in the setting of cardiac bypass surgery (Table 1). Furthermore, a research collaboration between the UK (Hausenloy) and Denmark (Botker) will investigate the effect of limb RIC on improving clinical outcomes in STEMI patients treated by PPCI in the RIC-PPCI and CONDI2 trials (Table 1). Depending on the results of these large multi-center clinical outcome studies, there is the potential for limb RIC to change clinical practice.

## Summary and Conclusions

The 8th Biennial Hatter Cardiovascular Workshop provided a great opportunity to discuss recent developments in the research field of limb RIC including: (1) new insights into the mechanisms underlying limb RIC; (2) expansion of non-cardiac organ protection; (3) potentially novel clinical applications of limb RIC; and (4) an update of recently published and future clinical outcomes studies. Huge advances have clearly been made over the last few years regarding the mechanisms underlying limb RIC and its potential in the clinical setting, thereby enabling limb RIC to make the journey from an intriguing experimental observation to a clinical application for patient benefit.

## References

[1] Abdul-Ghani S, Heesom KJ, Angelini GD, Suleiman MS (2014). Cardiac phosphoproteomics during remote ischemic preconditioning: a role for the sarcomeric Z-disk proteins. Biomed Res Int.

[2] Abu-Amara M, Yang SY, Quaglia A, Rowley P, Fuller B, Seifalian A, Davidson B (2011). Role of endothelial nitric oxide synthase in remote ischemic preconditioning of the mouse liver. Liver Transpl.

[3] Abu-Amara M, Yang SY, Quaglia A, Rowley P, Tapuria N, Fuller B, Davidson B, Seifalian A (2012). The hepatic soluble guanylyl cyclase-cyclic guanosine monophosphate pathway mediates the protection of remote ischemic preconditioning on the microcirculation in liver ischemia-reperfusion injury. Transplantation.

[4] Ali ZA, Callaghan CJ, Lim E, Ali AA, Nouraei SA, Akthar AM, Boyle JR, Varty K, Kharbanda RK, Dutka DP, Gaunt ME (2007). Remote ischemic preconditioning reduces myocardial and renal injury after elective abdominal aortic aneurysm repair: a randomized controlled trial. Circulation.

[5] Azzopardi DV, Strohm B, Edwards AD, Dyet L, Halliday HL, Juszczak E, Kapellou O, Levene M, Marlow N, Porter E, Thoresen M, Whitelaw A, Brocklehurst P (2009). Moderate hypothermia to treat perinatal asphyxial encephalopathy. N Engl J Med.

[6] Basalay M, Barsukevich V, Mastitskaya S, Mrochek A, Pernow J, Sjoquist PO, Ackland GL, Gourine AV, Gourine A (2012). Remote ischaemic pre- and delayed postconditioning-similar degree of cardioprotection but distinct mechanisms. Exp Physiol.

[7] Bell RM, Rear R, Cunningham J, Dawnay A, Yellon DM (2014). Effect of remote ischaemic conditioning on contrast-induced nephropathy in patients undergoing elective coronary angiography (ERICCIN): rationale and study design of a randomised single-centre, double-blind placebo-controlled trial. Clin Res Cardiol.

[8] Botker HE, Kharbanda R, Schmidt MR, Bottcher M, Kaltoft AK, Terkelsen CJ, Munk K, Andersen NH, Hansen TM, Trautner S, Lassen JF, Christiansen EH, Krusell LR, Kristensen SD, Thuesen L, Nielsen SS, Rehling M, Sorensen HT, Redington AN, Nielsen TT (2010). Remote ischaemic conditioning before hospital admission, as a complement to angioplasty, and effect on myocardial salvage in patients with acute myocardial infarction: a randomised trial. Lancet.

[9] Burton JO, Jefferies HJ, Selby NM, McIntyre CW (2009). Hemodialysis-induced cardiac injury: determinants and associated outcomes. Clin J Am Soc Nephrol.

[10] Candilio L, Hausenloy DJ, Yellon DM (2011). Remote ischemic conditioning: a clinical trial’s update. J Cardiovasc Pharmacol Ther.

[11] Candilio L, Malik A, Ariti C, Barnard M, Di SC, Lawrence D, Hayward M, Yap J, Roberts N, Sheikh A, Kolvekar S, Hausenloy DJ, Yellon DM (2014). Effect of remote ischaemic preconditioning on clinical outcomes in patients undergoing cardiac bypass surgery: a randomised controlled clinical trial. Heart.

[12] Cheung MM, Kharbanda RK, Konstantinov IE, Shimizu M, Frndova H, Li J, Holtby HM, Cox PN, Smallhorn JF, Van Arsdell GS, Redington AN (2006). Randomized controlled trial of the effects of remote ischemic preconditioning on children undergoing cardiac surgery: first clinical application in humans. J Am Coll Cardiol.

[13] Contractor H, Stottrup NB, Cunnington C, Manlhiot C, Diesch J, Ormerod JO, Jensen R, Botker HE, Redington A, Schmidt MR, Ashrafian H, Kharbanda RK (2013). Aldehyde dehydrogenase-2 inhibition blocks remote preconditioning in experimental and human models. Basic Res Cardiol.

[14] Crimi G, Ferlini M, Gallo F, Sormani MP, Raineri C, Bramucci E, De Ferrari GM, Pica S, Marinoni B, Repetto A, Raisaro A, Leonardi S, Rubartelli P, Visconti LO, Ferrario M (2014). Remote ischemic postconditioning as a strategy to reduce acute kidney injury during primary PCI: a post hoc analysis of a randomized trial. Int J Cardiol.

[15] Crimi G, Pica S, Raineri C, Bramucci E, De Ferrari GM, Klersy C, Ferlini M, Marinoni B, Repetto A, Romeo M, Rosti V, Massa M, Raisaro A, Leonardi S, Rubartelli P, Oltrona VL, Ferrario M (2013). Remote ischemic post-conditioning of the lower limb during primary percutaneous coronary intervention safely reduces enzymatic infarct size in anterior myocardial infarction: a randomized controlled trial. JACC Cardiovasc Interv.

[16] Crowley LE, McIntyre CW (2013). Remote ischaemic conditioning-therapeutic opportunities in renal medicine. Nat Rev Nephrol.

[17] D’Ascenzo F, Cavallero E, Moretti C, Omede P, Sciuto F, Rahman IA, Bonser RS, Yunseok J, Wagner R, Freiberger T, Kunst G, Marber MS, Thielmann M, Ji B, Amr YM, Modena MG, Zoccai GB, Sheiban I, Gaita F (2012). Remote ischaemic preconditioning in coronary artery bypass surgery: a meta-analysis. Heart.

[18] D’Ascenzo F, Moretti C, Omede P, Cerrato E, Cavallero E, Er F, Presutti DG, Colombo F, Crimi G, Conrotto F, Dinicolantonio JJ, Chen S, Prasad A, Biondi ZG, Gaita F (2014). Cardiac remote ischaemic preconditioning reduces periprocedural myocardial infarction for patients undergoing percutaneous coronary interventions: a meta-analysis of randomised clinical trials. EuroIntervention.

[19] Davidson SM, Selvaraj P, He D, Boi-Doku C, Yellon RL, Vicencio JM, Yellon DM (2013). Remote ischaemic preconditioning involves signalling through the SDF-1alpha/CXCR4 signalling axis. Basic Res Cardiol.

[20] Davies WR, Brown AJ, Watson W, McCormick LM, West NE, Dutka DP, hoole SP (2013). Remote ischemic preconditioning improves outcome at 6 years after elective percutaneous coronary intervention: the crisp stent trial long-term follow-up. Circ Cardiovasc Interv.

[21] Dickson EW, Reinhardt CP, Renzi FP, Becker RC, Porcaro WA, Heard SO (1999). Ischemic preconditioning may be transferable via whole blood transfusion: preliminary evidence. J Thromb Thrombolysis.

[22] Donato M, Buchholz B, Rodriguez M, Perez V, Inserte J, Garcia-Dorado D, Gelpi RJ (2013). Role of the parasympathetic nervous system in cardioprotection by remote hindlimb ischaemic preconditioning. Exp Physiol.

[23] Dong JH, Liu YX, Ji ES, He RR (2004). Limb ischemic preconditioning reduces infarct size following myocardial ischemia-reperfusion in rats. Sheng Li Xue Bao.

[24] Er F, Nia AM, Dopp H, Hellmich M, Dahlem KM, Caglayan E, Kubacki T, Benzing T, Erdmann E, Burst V, Gassanov N (2012). Ischemic preconditioning for prevention of contrast medium-induced nephropathy: randomized pilot RenPro Trial (Renal Protection Trial). Circulation.

[25] Ferdinandy P, Hausenloy DJ, Heusch G, Baxter GF, Schulz R (2014). Interaction of risk factors, comorbidities, and comedications with ischemia/reperfusion injury and cardioprotection by preconditioning, postconditioning, and remote conditioning. Pharmacol Rev.

[26] Gassanov N, Nia AM, Caglayan E, Er F (2014). Remote ischemic preconditioning and renoprotection: from myth to a novel therapeutic option?. J Am Soc Nephrol.

[27] Gho BC, Schoemaker RG, van den Doel MA, Duncker DJ, Verdouw PD (1996). Myocardial protection by brief ischemia in noncardiac tissue. Circulation.

[28] Giricz Z, Varga ZV, Baranyai T, Sipos P, Paloczi K, Kittel A, Buzas EI, Ferdinandy P (2014). Cardioprotection by remote ischemic preconditioning of the rat heart is mediated by extracellular vesicles. J Mol Cell Cardiol.

[29] Hahn CD, Manlhiot C, Schmidt MR, Nielsen TT, Redington AN (2011). Remote ischemic per-conditioning: a novel therapy for acute stroke?. Stroke.

[30] Hajrasouliha AR, Tavakoli S, Ghasemi M, Jabehdar-Maralani P, Sadeghipour H, Ebrahimi F, Dehpour AR (2008). Endogenous cannabinoids contribute to remote ischemic preconditioning via cannabinoid CB2 receptors in the rat heart. Eur J Pharmacol.

[31] Hausenloy DJ (2013). Cardioprotection techniques: preconditioning, postconditioning and remote conditioning (basic science). Curr Pharm Des.

[32] Hausenloy DJ, Baxter G, Bell R, Botker HE, Davidson SM, Downey J, Heusch G, Kitakaze M, Lecour S, Mentzer R, Mocanu MM, Ovize M, Schulz R, Shannon R, Walker M, Walkinshaw G, Yellon DM (2010). Translating novel strategies for cardioprotection: the Hatter Workshop Recommendations. Basic Res Cardiol.

[33] Hausenloy DJ, Candilio L, Laing C, Kunst G, Pepper J, Kolvekar S, Evans R, Robertson S, Knight R, Ariti C, Clayton T, Yellon DM (2012). Effect of remote ischemic preconditioning on clinical outcomes in patients undergoing coronary artery bypass graft surgery (ERICCA): rationale and study design of a multi-centre randomized double-blinded controlled clinical trial. Clin Res Cardiol.

[34] Hausenloy DJ, Erik BH, Condorelli G, Ferdinandy P, Garcia-Dorado D, Heusch G, Lecour S, van Laake LW, Madonna R, Ruiz-Meana M, Schulz R, Sluijter JP, Yellon DM, Ovize M (2013). Translating cardioprotection for patient benefit: position paper from the Working Group of Cellular Biology of the Heart of the European Society of Cardiology. Cardiovasc Res.

[35] Hausenloy DJ, Mwamure PK, Venugopal V, Harris J, Barnard M, Grundy E, Ashley E, Vichare S, Di Salvo C, Kolvekar S, Hayward M, Keogh B, MacAllister RJ, Yellon DM (2007). Effect of remote ischaemic preconditioning on myocardial injury in patients undergoing coronary artery bypass graft surgery: a randomised controlled trial. Lancet.

[36] Hausenloy DJ, Yellon DM (2004). New directions for protecting the heart against ischaemia-reperfusion injury: targeting the Reperfusion Injury Salvage Kinase (RISK)-pathway. Cardiovasc Res.

[37] Hausenloy DJ, Yellon DM (2007). Reperfusion injury salvage kinase signalling: taking a RISK for cardioprotection. Heart Fail Rev.

[38] Hausenloy DJ, Yellon DM (2008). Remote ischaemic preconditioning: underlying mechanisms and clinical application. Cardiovasc Res.

[39] Heusch G (2013). Cardioprotection: chances and challenges of its translation to the clinic. Lancet.

[40] Heusch G, Schulz R (2002). Remote preconditioning. J Mol Cell Cardiol.

[41] Hibert P, Prunier-Mirebeau D, Beseme O, Chwastyniak M, Tamareille S, Lamon D, Furber A, Pinet F, Prunier F (2013). Apolipoprotein a-I is a potential mediator of remote ischemic preconditioning. PLoS One.

[42] Hong DM, Lee EH, Kim HJ, Min JJ, Chin JH, Choi DK, Bahk JH, Sim JY, Choi IC, Jeon Y (2014). Does remote ischaemic preconditioning with postconditioning improve clinical outcomes of patients undergoing cardiac surgery? Remote ischaemic preconditioning with postconditioning outcome trial. Eur Heart J.

[43] Hoole S, Heck PM, Sharples L, Khan SN, Duehmke R, Densem CG, Clarke SC, Shapiro LM, Schofield PM, O’Sullivan M, Dutka DP (2009). Cardiac Remote Ischemic Preconditioning in Coronary Stenting (CRISP Stent) Study: a prospective, randomized control trial. Circulation.

[44] Hougaard KD, Hjort N, Zeidler D, Sorensen L, Norgaard A, Thomsen RB, Jonsdottir K, Mouridsen K, Hansen TM, Cho TH, Nielsen TT, Botker HE, Ostergaard L, Andersen G (2013). Remote ischemic perconditioning in thrombolysed stroke patients: randomized study of activating endogenous neuroprotection-design and MRI measurements. Int J Stroke.

[45] Iliodromitis EK, Kyrzopoulos S, Paraskevaidis IA, Kolocassides KG, Adamopoulos S, Karavolias G, Kremastinos DT (2006). Increased C reactive protein and cardiac enzyme levels after coronary stent implantation. Is there protection by remote ischaemic preconditioning?. Heart.

[46] Jensen RV, Stottrup NB, Kristiansen SB, Botker HE (2012). Release of a humoral circulating cardioprotective factor by remote ischemic preconditioning is dependent on preserved neural pathways in diabetic patients. Basic Res Cardiol.

[47] Jones WK, Fan GC, Liao S, Zhang JM, Wang Y, Weintraub NL, Kranias EG, Schultz JE, Lorenz J, Ren X (2009). Peripheral nociception associated with surgical incision elicits remote nonischemic cardioprotection via neurogenic activation of protein kinase C signaling. Circulation.

[48] Kalakech H, Tamareille S, Pons S, Godin-Ribuot D, Carmeliet P, Furber A, Martin V, Berdeaux A, Ghaleh B, Prunier F (2013). Role of hypoxia inducible factor-1alpha in remote limb ischemic preconditioning. J Mol Cell Cardiol.

[49] Kanoria S, Jalan R, Davies NA, Seifalian AM, Williams R, Davidson BR (2006). Remote ischaemic preconditioning of the hind limb reduces experimental liver warm ischaemia-reperfusion injury. Br J Surg.

[50] Kant R, Diwan V, Jaggi AS, Singh N, Singh D (2008). Remote renal preconditioning-induced cardioprotection: a key role of hypoxia inducible factor-prolyl 4-hydroxylases. Mol Cell Biochem.

[51] Karuppasamy P, Chaubey S, Dew T, Musto R, Sherwood R, Desai J, John L, Shah AM, Marber MS, Kunst G (2011). Remote intermittent ischemia before coronary artery bypass graft surgery: a strategy to reduce injury and inflammation?. Basic Res Cardiol.

[52] Kingma JG, Simard D, Voisine P, Rouleau JR (2011). Role of the autonomic nervous system in cardioprotection by remote preconditioning in isoflurane-anaesthetized dogs. Cardiovasc Res.

[53] Kleinbongard P, Thielmann M, Jakob H, Peters J, Heusch G, Kottenberg E (2013). Nitroglycerin does not interfere with protection by remote ischemic preconditioning in patients with surgical coronary revascularization under isoflurane anesthesia. Cardiovasc Drugs Ther.

[54] Korsheed S, Crowley LE, Fluck RJ, McIntyre CW (2013). Creation of an arteriovenous fistula is associated with significant acute local and systemic changes in microvascular function. Nephron Clin Pract.

[55] Kottenberg E, Musiolik J, Thielmann M, Jakob H, Peters J, Heusch G (2014). Interference of propofol with signal transducer and activator of transcription 5 activation and cardioprotection by remote ischemic preconditioning during coronary artery bypass grafting. J Thorac Cardiovasc Surg.

[56] Kottenberg E, Thielmann M, Bergmann L, Heine T, Jakob H, Heusch G, Peters J (2012). Protection by remote ischemic preconditioning during coronary artery bypass graft surgery with isoflurane but not propofol–a clinical trial. Acta Anaesthesiol Scand.

[57] Kottenberg E, Thielmann M, Kleinbongard P, Frey UH, Heine T, Jakob H, Heusch G, Peters J (2014). Myocardial protection by remote ischaemic pre-conditioning is abolished in sulphonylurea-treated diabetics undergoing coronary revascularisation. Acta Anaesthesiol Scand.

[58] Lang SC, Elsasser A, Scheler C, Vetter S, Tiefenbacher CP, Kubler W, Katus HA, Vogt AM (2006). Myocardial preconditioning and remote renal preconditioning–identifying a protective factor using proteomic methods?. Basic Res Cardiol.

[59] Lee AC, Kozuki N, Blencowe H, Vos T, Bahalim A, Darmstadt GL, Niermeyer S, Ellis M, Robertson NJ, Cousens S, Lawn JE (2013). Intrapartum-related neonatal encephalopathy incidence and impairment at regional and global levels for 2010 with trends from 1990. Pediatr Res.

[60] Li J, Rohailla S, Gelber N, Rutka J, Sabah N, Gladstone RA, Wei C, Hu P, Kharbanda RK, Redington AN (2014). MicroRNA-144 is a circulating effector of remote ischemic preconditioning. Basic Res Cardiol.

[61] Li J, Xuan W, Yan R, Tropak MB, Jean-St-Michel E, Liang W, Gladstone R, Backx PH, Kharbanda RK, Redington AN (2011). Remote preconditioning provides potent cardioprotection via PI3K/Akt activation and is associated with nuclear accumulation of beta-catenin. Clin Sci (Lond).

[62] Liem DA, Verdouw PD, Ploeg H, Kazim S, Duncker DJ (2002). Sites of action of adenosine in interorgan preconditioning of the heart. Am J Physiol Heart Circ Physiol.

[63] Lim SY, Yellon DM, Hausenloy DJ (2010). The neural and humoral pathways in remote limb ischemic preconditioning. Basic Res Cardiol.

[64] Mastitskaya S, Marina N, Gourine A, Gilbey MP, Spyer KM, Teschemacher AG, Kasparov S, Trapp S, Ackland GL, Gourine AV (2012). Cardioprotection evoked by remote ischaemic preconditioning is critically dependent on the activity of vagal pre-ganglionic neurones. Cardiovasc Res.

[65] McCrindle BW, Clarizia NA, Khaikin S, Holtby HM, Manlhiot C, Schwartz SM, Caldarone CA, Coles JG, Van Arsdell GS, Scherer SW, Redington AN (2014) Remote ischemic preconditioning in children undergoing cardiac surgery with cardiopulmonary bypass: a single-center double-blinded randomized trial. J Am Heart Assoc 3(4). doi:10.1161/JAHA.114.000964 10.1161/JAHA.114.000964 PMC4310383 25074698

[66] McDonald MA, Braga JR, Li J, Manlhiot C, Ross HJ, Redington AN (2014). A randomized pilot trial of remote ischemic preconditioning in heart failure with reduced ejection fraction. PLoS One.

[67] Meng R, Asmaro K, Meng L, Liu Y, Ma C, Xi C, Li G, Ren C, Luo Y, Ling F, Jia J, Hua Y, Wang X, Ding Y, Lo EH, Ji X (2012). Upper limb ischemic preconditioning prevents recurrent stroke in intracranial arterial stenosis. Neurology.

[68] Merlocco AC, Redington KL, Disenhouse T, Strantzas SC, Gladstone R, Wei C, Tropak MB, Manlhiot C, Li J, Redington AN (2014). Transcutaneous electrical nerve stimulation as a novel method of remote preconditioning: in vitro validation in an animal model and first human observations. Basic Res Cardiol.

[69] Meybohm P, Zacharowski K, Cremer J, Roesner J, Kletzin F, Schaelte G, Felzen M, Strouhal U, Reyher C, Heringlake M, Schon J, Brandes I, Bauer M, Knuefermann P, Wittmann M, Hachenberg T, Schilling T, Smul T, Maisch S, Sander M, Moormann T, Boening A, Weigand MA, Laufenberg R, Werner C, Winterhalter M, Treschan T, Stehr SN, Reinhart K, Hasenclever D, Brosteanu O, Bein B (2012). Remote ischaemic preconditioning for heart surgery. The study design for a multi-center randomized double-blinded controlled clinical trial–the RIPHeart-Study. Eur Heart J.

[70] Moretti C, Cavallero E, D’Ascenzo F, Cerrato E, Zoccai GB, Omede P, Presutti DG, Lefevre T, Sanguineti F, Picchi A, Palazzuoli A, Carini G, Giammaria M, Ugo F, Presbitero P, Chen S, Lin S, Sheiban I, Gaita F (2014). The EUROpean and Chinese cardiac and renal Remote Ischemic Preconditioning Study (EURO-CRIPS): study design and methods. J Cardiovasc Med (Hagerstown).

[71] Murry CE, Jennings RB, Reimer KA (1986). Preconditioning with ischemia: a delay of lethal cell injury in ischemic myocardium. Circulation.

[72] Ovize M, Baxter GF, Di Lisa F, Ferdinandy P, Garcia-Dorado D, Hausenloy DJ, Heusch G, Vinten-Johansen J, Yellon DM, Schulz R (2010). Postconditioning and protection from reperfusion injury: where do we stand? Position paper from the Working Group of Cellular Biology of the Heart of the European Society of Cardiology. Cardiovasc Res.

[73] Patel HH, Moore J, Hsu AK, Gross GJ (2002). Cardioprotection at a distance: mesenteric artery occlusion protects the myocardium via an opioid sensitive mechanism. J Mol Cell Cardiol.

[74] Polkinghorne KR, McDonald SP, Atkins RC, Kerr PG (2004). Vascular access and all-cause mortality: a propensity score analysis. J Am Soc Nephrol.

[75] Prunier F, Angoulvant D, Saint EC, Vermes E, Gilard M, Piot C, Roubille F, Elbaz M, Ovize M, Biere L, Jeanneteau J, Delepine S, Benard T, Abi-Khalil W, Furber A (2014). The RIPOST-MI study, assessing remote ischemic perconditioning alone or in combination with local ischemic postconditioning in ST-segment elevation myocardial infarction. Basic Res Cardiol.

[76] Przyklenk K, Bauer B, Ovize M, Kloner RA, Whittaker P (1993). Regional ischemic ‘preconditioning’ protects remote virgin myocardium from subsequent sustained coronary occlusion. Circulation.

[77] Rahman IA, Mascaro JG, Steeds RP, Frenneaux MP, Nightingale P, Gosling P, Townsend P, Townend JN, Green D, Bonser RS (2010). Remote ischemic preconditioning in human coronary artery bypass surgery: from promise to disappointment?. Circulation.

[78] Rassaf T, Ferdinandy P, Schulz R (2014). Nitrite in organ protection. Br J Pharmacol.

[79] Rassaf T, Totzeck M, Hendgen-Cotta UB, Shiva S, Heusch G, Kelm M (2014). Circulating nitrite contributes to cardioprotection by remote ischemic preconditioning. Circ Res.

[80] Redington KL, Disenhouse T, Li J, Wei C, Dai X, Gladstone R, Manlhiot C, Redington AN (2013). Electroacupuncture reduces myocardial infarct size and improves post-ischemic recovery by invoking release of humoral, dialyzable, cardioprotective factors. J Physiol Sci.

[81] Redington KL, Disenhouse T, Strantzas SC, Gladstone R, Wei C, Tropak MB, Dai X, Manlhiot C, Li J, Redington AN (2012). Remote cardioprotection by direct peripheral nerve stimulation and topical capsaicin is mediated by circulating humoral factors. Basic Res Cardiol.

[82] Rentoukas I, Giannopoulos G, Kaoukis A, Kossyvakis C, Raisakis K, Driva M, Panagopoulou V, Tsarouchas K, Vavetsi S, Pyrgakis V, Deftereos S (2010). Cardioprotective role of remote ischemic periconditioning in primary percutaneous coronary intervention: enhancement by opioid action. JACC Cardiovasc Interv.

[83] Schwartz LL, Kloner RA, Arai AE, Baines CP, Bolli R, Braunwald E, Downey J, Gibbons RJ, Gottlieb RA, Heusch G, Jennings RB, Lefer DJ, Mentzer RM, Murphy E, Ovize M, Ping P, Przyklenk K, Sack MN, Vander Heide RS, Vinten-Johansen J, Yellon DM (2011). New horizons in cardioprotection: recommendations from the 2010 national heart, lung, and blood institute workshop. Circulation.

[84] Shimizu M, Tropak M, Diaz RJ, Suto F, Surendra H, Kuzmin E, Li J, Gross G, Wilson GJ, Callahan J, Redington AN (2009). Transient limb ischaemia remotely preconditions through a humoral mechanism acting directly on the myocardium: evidence suggesting cross-species protection. Clin Sci (Lond).

[85] Sloth AD, Schmidt MR, Munk K, Kharbanda RK, Redington AN, Schmidt M, Pedersen L, Sorensen HT, Botker HE (2014). Improved long-term clinical outcomes in patients with ST-elevation myocardial infarction undergoing remote ischaemic conditioning as an adjunct to primary percutaneous coronary intervention. Eur Heart J.

[86] Steensrud T, Li J, Dai X, Manlhiot C, Kharbanda RK, Tropak M, Redington A (2010). Pretreatment with the nitric oxide donor SNAP or nerve transection blocks humoral preconditioning by remote limb ischemia or intra-arterial adenosine. Am J Physiol Heart Circ Physiol.

[87] Tang ZL, Dai W, Li YJ, Deng HW (1999). Involvement of capsaicin-sensitive sensory nerves in early and delayed cardioprotection induced by a brief ischaemia of the small intestine. Naunyn Schmiedebergs Arch Pharmacol.

[88] Tehrani S, Laing C, Yellon DM, Hausenloy DJ (2013). Contrast-induced acute kidney injury following PCI. Eur J Clin Invest.

[89] Thielmann M, Kottenberg E, Boengler K, Raffelsieper C, Neuhaeuser M, Peters J, Jakob H, Heusch G (2010). Remote ischemic preconditioning reduces myocardial injury after coronary artery bypass surgery with crystalloid cardioplegic arrest. Basic Res Cardiol.

[90] Thielmann M, Kottenberg E, Kleinbongard P, Wendt D, Gedik N, Pasa S, Price V, Tsagakis K, Neuhauser M, Peters J, Jakob H, Heusch G (2013). Cardioprotective and prognostic effects of remote ischaemic preconditioning in patients undergoing coronary artery bypass surgery: a single-centre randomised, double-blind, controlled trial. Lancet.

[91] Venugopal V, Laing CM, Ludman A, Yellon DM, Hausenloy D (2010). Effect of remote ischemic preconditioning on acute kidney injury in nondiabetic patients undergoing coronary artery bypass graft surgery: a secondary analysis of 2 small randomized trials. Am J Kidney Dis.

[92] Walsh SR, Boyle JR, Tang TY, Sadat U, Cooper DG, Lapsley M, Norden AG, Varty K, Hayes PD, Gaunt ME (2009). Remote ischemic preconditioning for renal and cardiac protection during endovascular aneurysm repair: a randomized controlled trial. J Endovasc Ther.

[93] Wei M, Xin P, Li S, Tao J, Li Y, Li J, Liu M, Li J, Zhu W, Redington AN (2011). Repeated remote ischemic postconditioning protects against adverse left ventricular remodeling and improves survival in a rat model of myocardial infarction. Circ Res.

[94] White SK, Frohlich GM, Sado DM, Maestrini V, Fontana M, Treibel TA, Tehrani S, Flett AS, Meier P, Ariti C, Davies JR, Moon JC, Yellon DM, Hausenloy DJ (2014). Remote ischemic conditioning reduces myocardial infarct size and edema in patients with st-segment elevation myocardial infarction. JACC Cardiovasc Interv.

[95] Wolfrum S, Schneider K, Heidbreder M, Nienstedt J, Dominiak P, Dendorfer A (2002). Remote preconditioning protects the heart by activating myocardial PKCepsilon-isoform. Cardiovasc Res.

